# Spirometry practice by French general practitioners between 2010 and 2018 in adults aged 40 to 75 years

**DOI:** 10.1038/s41533-023-00352-9

**Published:** 2023-09-30

**Authors:** A. Chapron, T. Lemée, G. Pau, S. Jouneau, S. Kerbrat, F. Balusson, E. Oger

**Affiliations:** 1 Rennes University, Centre Hospitalier Universitaire (CHU) de Rennes, Department of General Practice, Rennes, France; 2 Rennes University, Centre Hospitalier Universitaire (CHU) de Rennes, Institut National de la Santé et de la Recherche Médicale (INSERM), CIC-1414 Rennes, France; 3 Rennes University, CHU Rennes, INSERM, Ecole des hautes études en santé publique (EHESP), Institut de recherche en santé, environnement et travail (IRSET) - UMR_S 1085, Rennes, France; 4 CHU Rennes, Department of Respiratory Medicine, Rennes, France

**Keywords:** Health care, Medical research

## Abstract

In France, most spirometries are performed by pneumologists. Spirometry is difficult to access due to the distance to medical office and long delays for appointments. This lack of accessibility contributes to the underdiagnosis of chronic obstructive pulmonary disease (COPD) among patients aged between 40 and 75 years. In recent years, general practitioners (GPs) have been performing spirometry in private practice. However, the extent of this practice is unknown. A French retrospective, repetitive transversal study analysed data from the “Système National des Données de Santé” (SNDS) database. The targeted population was GPs in primary care that performed spirometries between 2010 and 2018, in patients aged between 40 and 75 years. Between 2010 and 2018, 302,674 (7.2%) spirometries were performed in France by GPs in private practices, in patients 40 to 75 years old. 5.4% by “expert GPs” (>60 spirometries/year) and 1.8% by “non-expert GPs”. In “non-expert GPs” (2.8% of French GPs in 2018), the annual number of spirometries increased by 701 each year (*p* < 2.10^4^), the annual number of GPs performing spirometries increased by 114 each year (*p* < 2.10^−5^). Overall, 24.9% of the spirometries performed by GPs were referrals from other GPs. The number of spirometries performed by GPs and the number of GPs performing spirometries has gradually increased over time. However, this increase is inadequate considering the need to early detect and follow up respiratory disorders.

## Introduction

Spirometry is used to examine respiratory function and allows early diagnosis and follow-up of obstructive respiratory conditions, including asthma and Chronic Obstructive Pulmonary Disease (COPD). Depending on the country and its healthcare system, spirometry is performed by different healthcare professionals^1^. In the USA and in most European countries, spirometry is predominantly performed by primary healthcare professionals, such as General Practitioners (GPs) and nurses. While in France, spirometry is almost exclusively performed by pneumologists after referrals by GPs^2^.

In France, about 19% of consultation with GPs concern respiratory problems that are often infectious. Among these consultations, only 4.5% are follow-up visits for patients with asthma or COPD^3^. These consultations represent an opportunity for GPs to suggest and perform spirometries.

COPD is a common chronic obstructive respiratory condition occurring in an estimated 3.5 million people in France: two-thirds of them are not aware that they have COPD^4^. In France, COPD prevalence data are scarce since epidemiological studies rarely have access to spirometry data. Since 2003, the “Fédération Française de Pneumologie” (FFP) and the “Société Française de Pneumologie de Langue Française” (SPLF) have emphasised the important role of GPs for early diagnosis of COPD, especially considering the under-diagnosis of COPD for adults between the ages of 40 and 75, a common age cut-off in studies assessing early detection of COPD.

The Global Initiative for Chronic Obstructive Lung Disease (GOLD) recommends that spirometry be performed in symptomatic patients exposed to at least one risk factor: history of smoking, occupational exposure, and pollution^5–7^. In the United Kingdom, the National Institute for Health and Care Excellence (NICE) recommends COPD screening with spirometry in people older than 35 years of age, who smoke or who have smoked, and with one of the following symptoms: chronic cough, exertional breathlessness, regular sputum production, frequent winter “bronchitis”, and wheezing^8^. This screening can be performed by various healthcare professionals that includes GPs, physiotherapists, and nurses specialised in pneumology. In France, guidelines for management of COPD from the French health authority, the “Haute Autorité de Santé (HAS)”, states that COPD diagnosis be based on evidence of an obstructive ventilatory disorder, by standard spirometry, with bronchodilator reversibility testing, in adults older than 40 years and with at least one risk factor^9^.

Approximately, 2 million French have undiagnosed COPD^10^. The need to detect and diagnose COPD, as early as possible during disease evolution, is largely unmet. Moreover, the number of registered pneumologist in France, <3000^11^, are insufficient to meet this healthcare need. Spirometry is difficult to access due to the distance to medical office and long delays for appointments. For many years, French GPs have been allowed to detect COPD, performing spirometry as part of their practice. Specific training in spirometry allows GPs to develop these skills for primary care, thus facilitating patient access to spirometry. These spirometries performed by GPs, in primary care, also allow them to follow-up diseases, optimise treatment, and to explore symptoms in patients with respiratory diseases^12^. However, the extent of this practice is unknown. This study is part of an initiative to assess the extent to which French GPs perform spirometries. The objective was to describe the practice of spirometry by French GPs between 2010 and 2018, in patients aged 40 to 75 years.

## Methods

The study was designed to retrospectively analyse data from the national “Système National d’Information Inter Régimes de l’Assurance maladie” (SNIIRAM), now renamed the “Système National des Données de Santé” (SNDS). The data analysed concerned spirometries performed by GPs in private practice. No patients were involved in this study.

### The SNDS database

The SNDS database comprises databases that collect data concerning medical and administrative healthcare resources reimbursed in private practice and in hospitals. The SNDS is managed by the “Caisse Nationale d’Assurance Maladie” (CNAM)^13^ where the data from almost all the 64 million French people in 2018 are stored. In the SNDS database, the following individual pseudonymised data from patients that used healthcare resources are available: demographic data (age, gender, and residential city, town, etc.), healthcare insurance coverage, chronic illnesses, use of ambulatory healthcare resources (medical acts, medications reimbursed etc.), and hospital data (duration of hospitalisation, diagnosis according to the International Classification of Diseases, 10^th^ edition [ICD-10]).

### Selection of general practitioners for the study

The target population was general practitioners (GPs) in primary practice that performed at least 1 spirometry between the 1st of January 2010 and the 31st of December 2018 and for patients aged between 40 and 75 years. This age group of patients is most often considered in national and international guidelines and most often included in studies investigating early detection of COPD in primary care^14^. In France, GPs can practice in a variety of healthcare structures: private practice, medico-social, and/or hospitals (private and public sectors). Moreover, GPs can specialise within their practice, specialities including allergology, angiology, gerontology, and sports medicine. In the SNDS database, GPs are coded in the SNDS as either 1, 22, or 23, according to how they obtained their GP qualification. These codes group diverse GPs with various qualifications and medical specialisations, types of practices (private practices alone or combined with other practices), locations of practices (urban or rural), and with divergent patient profiles (in terms of demographics and healthcare needs). In our study, we were interested in GPs in private practice (family doctors), treating patients of all ages consulting for diverse medical conditions, that performed spirometry. GPs practicing in hospitals and those with a speciality, coded in the SNDS, were not assessed during this study. However, the study did not want to analyse GPs that performed an exceptional number of spirometries since these GPs do not represent most GPs. Their inclusion in the analysis would bias the sampling. This study is part of an initiative to establish whether the majority of French GPs should be encouraged to perform spirometries. The median annual number of spirometries performed by GPs were calculated for the years 2010 to 2018. In this study, we wanted to focus on “non-expert GPs”, as they are the core target for a public health policy that aims to strengthen the practice of spirometry in primary care in France. Thus, the global median of annual medians was calculated to be 6 spirometries per year. GPs performing >60 spirometries per year ( > 10 times the median, corresponding to the 90^th^ percentile of the annual spirometries performed (see Supplementary Information 1), were considered as “expert GPs” performing an exceptional number of spirometries. The remaining “non-expert GPs” performed ≤60 spirometries per year were considered as our study population of interest.

### Extraction of data from the SNDS database

In our study, we extracted data from the SNDS database corresponding with patients treated by GPs in our target population, between 2010 and 2018, reimbursed for a standard spirometry: measuring of slow vital capacity (SVC) and forced vital capacity (FVC) with the data recorded, coded as GLQP012 in the SNDS. We only extracted data concerning medical acts performed by the targeted GPs. The study data were extracted on the 1^st^ of June 2020 by the CNAM.

### Statistical analysis

Statistical analysis of changes in the use of spirometry was performed using linear regression analysis, with the statistical software R. Linear modelling between variables, in our study, number of spirometries or number of GPs, and year yielded parameter estimates that we tested for significance. To calculate the proportion of French GPs performing spirometry between 2010 and 2018 we extracted the number of French GPs practicing during these years from the public Score Santé database on the 8^th^ of March 2021^15^. The database indicates the numbers of GPs practicing on the 1^st^ of January of each year. If a GP indicated several specialities in the database, only those that indicated general medicine as their primary speciality were considered. For the years, 2010 and 2011, the numbers of practicing GPs were not available. These data were estimated taking into account the annual decline in the number of GPs as indicated in the “Atlas national annuel de démographie médicale”^16^. The data are described annually, from 2010 to 2018, using descriptive statistics.

### Ethical and regulatory considerations

The CNAM created a dedicated and secure database for the study management team: the REcherches en Pharmaco-Epidémiologie et REcours aux Soins (REPERES) team at the Université de Rennes. The study has been authorised by the French regulatory body: l’Agence Nationale de Sécurité du Médicament et des produits de santé (ANSM). The study was conducted in partnership with the CNAM.

## Results

**Fig. 1:**
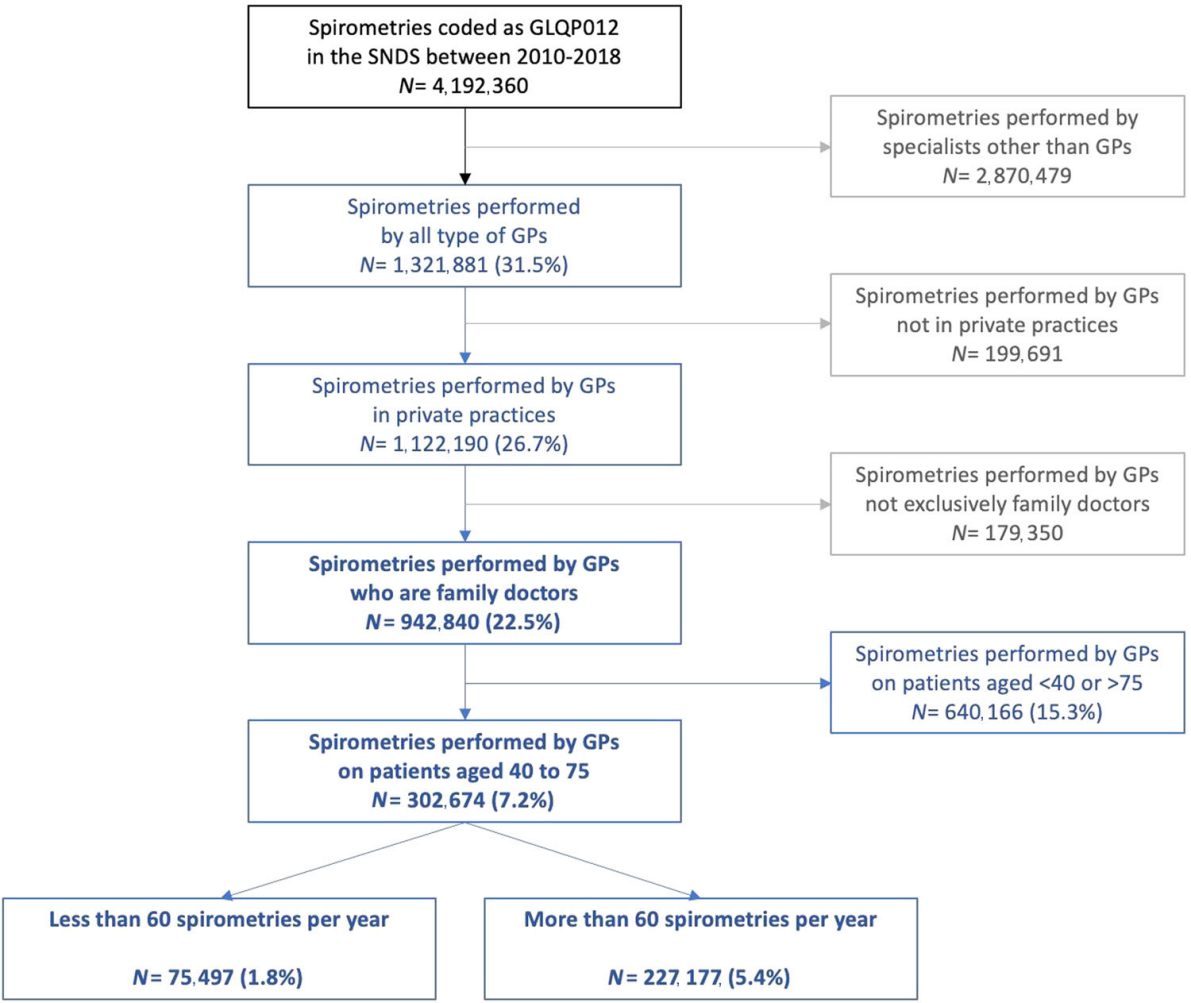
Spirometries performed by French general practitioners (GPs) between 2010 and 2018.

From 2010 to 2018, 4,192,360 spirometries were performed in France: 1,321,881 (31.5%) by GPs (coded 1, 22, or 23), irrespective of the type of healthcare structure (Fig. 1). Between 2010 and 2018, GPs primarily doing family medicine in private practice performed 942,840 (22.5%) spirometries of which 302,674 (7.2%) were performed in patients aged between 40 and 75 years. 227,177 (5.4%) by “expert GPs performing >60 spirometries per year and 75,497 (1.8%) by “non-expert GPs” performing ≤60 spirometries per year.

Most spirometries were carried out by a minority of “expert GPs”, performing >60 spirometries per year: 307 GPs in 2018, i.e., 0.5% of French GPs in 2018. The number of “expert GPs” did not increase significantly over the period under study: from 271 in 2010 to 307 in 2018. The following analyses focus on spirometries performed by “non-expert GPs”.

### Evolution of the spirometries performed by “non-expert” French GPs between 2010 and 2018 on patients aged 40 to 75 years

The annual number of spirometries performed by “non-expert GPs” gradually increased over the years, from 5572 performed in 2010 to 11,507 in 2018: a 106.5% increase (Table 1). This increase of about 701 new spirometries each year is significant (linear regression analysis, *p* < 2.10^−4^). These spirometries were performed on their patients and on those referred to them by other healthcare professionals. The annual number of spirometries performed by GPs for their patients increased from 3,479 in 2010 to 8,801 in 2018: an annual increase of 5,322 (153%). From 2010 and 2018, the annual number of spirometries performed by GPs for referred patients increased by 29.3%: from 2,093 in 2010 to 2,706 in 2018. Most of the spirometries performed were for the GP’s patients. The proportions of spirometries GPs performed for their patients among all the spirometries they performed gradually increased from 62.4% in 2010 to 76.5% in 2018. On average, between 2010 and 2018, 75.1% of the spirometries performed by GPs were for their patients and 24.9% for referred patients.

**Table 1:** Evolution of the 75,497 spirometries performed by “non-expert GPs” between 2010 and 2018.

	2010	2011	2012	2013	2014	2015	2016	2017	2018
Spirometries performed each year									
Total	5572	6113	8239	7545	7145	8894	9696	10786	11507
GPs’ patients	3479	4222	6283	5780	5541	7068	7629	8539	8801
Referred patients	2093	1891	1956	1765	1604	1826	2067	2247	2706
Rate of spirometries performed in GPs’ patients (%)	62.44	69.07	76.26	76.61	77.55	79.47	78.68	79.17	76.48
GPs that performed at least one spirometry each year									
Total	681	868	1109	1067	1082	1247	1388	1574	1683
Spirometries per GP	8.18	7.04	7.43	7.07	6.60	7.13	6.99	6.85	6.84
Proportion of GPs performing spirometries (%) * (n)* = *(number of GPs in French private practice*^a^*)*	1.02 *(66 722)*	1.32 *(65 654)*	1.72 *(64 603)*	1.68 *(63 569)*	1.72 *(62 959)*	2.00 *(62 186)*	2.27 *(61 277)*	2.61 *(60 294)*	2.80 *(60 188)*
Patients that had spirometries performed by GPs									
Total	5145	5763	7745	7082	6754	8449	9288	10345	10976
Spirometries per patient									
Mean [min-max] Median	1.08 [1–14] 1	1.06 [1–13] 1	1.06 [1–12] 1	1.07 [1–13] 1	1.06 [1–13] 1	1.05 [1–5] 1	1.04 [1–5] 1	1.04 [1–5] 1	1.05 [1–7] 1

*GPs* general practitioners.

^a^Data from the Score Santé database: number of GPs in private practice in France for the given year^15^.

Between 2010 and 2018, the number of “non-expert GPs” performing spirometries increased from 681 (1.02% of GPs) in 2010 to 1,683 (2.80%) in 2018, a significant increase of about 114.4 new GPs each year (linear regression analysis, *p* < 2.10^−5^). This is despite the overall number of French GPs decreasing by 6534, a decrease of 9.8%, between 2010 and 2018.

In 2018, 10,976 patients had spirometries compared to 5145, in 2010: an increase of 113%. During the study period, the mean number of spirometries performed by GP decreased from 8.18 to 6.84. Also, the rate of patients, aged between 40 and 75 years, that had a spirometry performed by a GP increased from 19.45 to 38.42 for 100,000 inhabitants (see Supplementary Information 2).

### Profile of patients, aged 40–75 years, that had spirometries performed by “non-expert GPs” between 2010 and 2018

Between 2010 and 2018, the mean age of patients performing spirometry increased from 56.2 years in 2010 to 57.8 years in 2018 (Table 2). The gender ratio remained almost unchanged over the years, at about 50% women (ranging from 48.9% to 52.6%). The proportion of patients with chronic diseases has remained constant over the years. However, the proportion of patients for whom an affection of long duration (ALD), either severe chronic respiratory disorders or severe persistent asthma, was declared gradually declined between 2010 and 2018.

**Table 2:** Profile of patients, aged 40 to 75 years, that had spirometries performed by “non-expert” French GPs between 2010 and 2018.

	2010	2011	2012	2013	2014	2015	2016	2017	2018
Gender									
Total	5145	5763	7745	7082	6754	8449	9288	10345	10976
Female (%)	2637 (51.3)	2816 (48.9)	3867 (49.9)	3567 (50.4)	3460 (51.2)	4373 (51.8)	4815 (51.8)	5311 (51.3)	5769 (52.6)
Age									
Mean (standard deviation)	56.2 ( ± 9.9)	56.2 ( ± 9.8)	56.5 ( ± 9.7)	56.5 ( ± 9.7)	57 ( ± 9.6)	57.4 ( ± 9.6)	57.5 ( ± 9.6)	57.7 ( ± 9.6)	57.8 ( ± 9.6)
Patients with chronic diseases, declared in the SNDS									
All chronic diseases (i.e., the 30 ALD^a^ diseases recognised and covered by the CNAM) (%)	2190 (42.6)	2610 (45.3)	3448 (44.5)	3070 (43.3)	2892 (42.8)	3640 (43.1)	4042 (43.5)	4408 (42.6)	4487 (40.9)
Severe chronic respiratory disorders (ALD no14^a^) (%)	419 (8.1)	422 (7.3)	527 (6.8)	472 (6.7)	416 (6.2)	575 (6.8)	594 (6.4)	600 (5.8)	580 (5.3)
Severe persistant asthma (sub-group within ALD no14^a^) (%)	207 (4.0)	206 (3.6)	214 (2.8)	190 (2.7)	157 (2.3)	212 (2.5)	221 (2.4)	210 (2.0)	190 (1.7)

*ALD* affection of long duration (translated from the French “affection de longue durée”), *CNAM* “Caisse Nationale d’Assurance Maladie”, *GP* general practitioner, *SNDS* “Système National des Données de Santé”.

^a^The ALD is a medico-administrative declaration made by the GP to the CNAM, enabling these patients to be reimbursed at 100%. When making this declaration, the GP undertakes to ensure that the clinical or biological criteria defining the disease are met. ALDs are referenced in the SNDS, but no further clinical details are available for respiratory diagnoses. Only the sub-category “severe persistent asthma” within ALD n°14 is identifiable in the SNDS.

## Discussion

This study is the first to access the extent to which spirometries are performed by French GPs in private practice. Our results show a gradual increase in the number of spirometries performed in patients aged between 40 and 75 years, between 2010 and 2018 by “non-expert GPs”: the GPs of interest for public health strategies to increase the number of spirometries performed in private practice. Indeed, between 2010 and 2018, both the number of spirometries performed by GPs and the number of GPs performing spirometries significantly increased. Thus, over time, more patients have had access to spirometry in French primary care.

Between 2010 to 2018, the proportion of spirometries performed per GP decreased (to 6.8 spirometries per GP in 2018), while the number of GPs performing spirometry increased (+147%), as did the number of spirometries performed each year (+106%). Thus, overall, more and more patients benefited per GP spirometry: from 5,145 in 2010 to 10,976 in 2018. The decrease in the proportion of spirometries performed per GPs could be explained by a “screening effect”. Indeed, GPs do not yearly screen all their patients, aged 40–75 years with COPD risk factors. However, yearly spirometry could be performed, by GPs, to follow up respiratory disease evolution, e.g., COPD and asthma, particularly when the pathology is not complex and not severe. The fact that the mean number of spirometries performed per patient exceeded 1 implies that GPs do not only perform spirometries for screening and diagnosis, but also for disease follow-up. However, it is noteworthy that certain patients performed excessive numbers of spirometries per year, e.g., the maximum number of spirometries performed by a patient in 2010 was 14. Although marginal, this could be due to coding errors by GPs when completing the SNDS database.

The more GPs perform spirometries the better the quality of the spirometries. Tollånes et al.^17^ reported that the more Norwegian GPs performed spirometries the better they interpreted the spirometry results. In our study, more and more “non-expert GPs” were practicing spirometry, but the annual volume was low (6.8 spirometries per GP in 2018): thus the quality of spirometry performed may be questionable. However, a recent study evaluated spirometries, performed by French GPs, for COPD detection and reported a 93% positive predictive value for spirometries performed by GPs compared to reference spirometries for confirmation of diagnosis^18^. The low volume of spirometries performed by GPs, that we report, does imply that GPs need to have a professional activity that is compatible with performing spirometries^19^. Indeed, to perform a spirometry a dedicated consultation is needed. This can be difficult, particularly considering the increased tension, particularly in rural areas, due to medical desertification. In this context, it is understandable that certain GPs do not want to be trained to perform spirometries and to integrate this procedure in their practices, this despite the public health need.

Even though the number of spirometries performed by GPs, for patients aged 40–75 years, has increased by 147% from 2010 to 2018, only 2.8% of French “non-expert GPs” performed spirometries in 2018. This is insufficient considering the need for both the early detection of COPD and the follow up of non-severe COPD and asthma, relative to the prevalence of these diseases. Moreover, when prescribed, a larger proportion of patients underwent spirometries when the spirometry was performed by GPs compared to pneumologists. Indeed, a French study evaluating professional practices showed that 89% of eligible patients (*n* = 184) agreed to have spirometry performed by their GPs for the early detection of COPD, and 66% performed this specific consultation. Among them, 82% were satisfied that their GPs prescribed and performed their spirometries^20^. Overall, a qualitative study, performed in patients following a routine spirometry test to confirm COPD diagnosis by their GPs, showed that patients were pleased with the spirometry in primary care^21^. In patients prescribed a spirometry, fewer patients perform spirometries when the delays for an appointment were long: delays are often shorter with GPs than with pneumologist. Indeed, GPs are often geographically easier to access and more availability than pneumologists^22^.

The study screening also highlights the 277,177 GPs, with expertise in spirometry, that perform more than 60 spirometries per year: between 1 and 2 spirometries per week. Currently, with the reorganisation of French primary care to develop multidisciplinary groups, it may be more pertinent to encourage GPs with expertise to perform more spirometries, instead of encouraging more GPs to perform few spirometries. We observed in our study that about 25% of patients with spirometries performed by GPs were referrals from other primary healthcare providers. Thus, in France, GPs without spirometry expertise are referring patients to GPs with this expertise. These referrals allow trained GPs to perform more spirometries and improves interpretation of results, and making spirometry more accessible for patients.

This is the first study to access the extent to which GPs performed spirometries in France, between 2010 and 2018, based on data from the SNDS. The patients included in our study are similar to those enrolled in studies accessing early detection of COPD in primary care^14,23,24^. The Score Santé database was used to obtain the annual numbers of GPs practicing in France. This database only concerns GPs in private practice. However, in the last few years, primary healthcare centres have developed that employ GPs. Therefore, using the data from the Score Santé database may overestimate the proportion of GPs performing spirometries. During the period under study (2010–2018), and at present, most GPs were in private practice. In contrast, the number of spirometries performed in 2017 and 2018 may be slightly underestimated. During these years, GPs that performed spirometries for referred patients could have coded the spirometry as an “exceptional consultation”, and not GLQP012, without needing to specify that it was spirometry. These spirometries would not have been extracted from the SNDS for our study. In the SNDS, the GLQP012 code does not specify whether the spirometry was performed for detecting or monitoring a respiratory pathology; nor does it indicate the pathology of interest (COPD, asthma, or another respiratory disorder). Notwithstanding, considering the age of our population (40 to 75 years old) and the increased prevalence of COPD, compared to asthma, in this age group, we suppose that most of the spirometries performed were to detect or follow up COPD. Finally, it is difficult to compare our data with those of other countries owing to the substantial differences in healthcare and primary care structures and organisations.

Our study shows that, in France, in patients 40–75 years old, the annual number of spirometries rose between 2010 and 2018, as did the number of non-expert GPs performing them. Furthermore, between 2010 and 2018, the number of patients undergoing spirometries increased. Currently, COPD is largely underdiagnosed. Early diagnosis of COPD, particularly in primary care, is essential. Although our study shows that more and more French GPs are performing spirometries, it is critical that GPs be encouraged to develop expertise to perform spirometries to detect respiratory disorders, particularly COPD, and to follow up respiratory diseases.

### Supplementary information

suplementary informtions

## Data Availability

The data supporting the results of this study are available from the corresponding author on reasonable request (secondary analysis data). Consultation of the raw source data is subject to the agreement of the CNAM.

## References

[1] Zysman M (2014). [COPD and perception of the new GOLD document in Europe. Workshop from the Societe de pneumologie de langue francaise (SPLF)]. Rev. Mal. Respir..

[2] Darmon D (2015). [Screening for COPD in general practice: which perspectives?]. Rev. Mal. Respir..

[3] Carron M (2015). ECOGEN RESPI : étude des résultats de consultation associés à un motif respiratoire en médecine générale. Exercer.

[4] Fuhrman C, Delmas MC, pour le groupe epidemiologie et recherche clinique de la SPLF. (2010). [Epidemiology of chronic obstructive pulmonary disease in France]. Rev. Mal. Respir..

[5] Global Initiative for Chronic Obstructive Lung Disease (GOLD). *Global Strategy For The Diagnosis, Management, And Prevention Of Chronic Obstructive Pulmonary Disease**(2022 report)*. (2021).

[6] Eisner MD (2010). An official American Thoracic Society public policy statement: novel risk factors and the global burden of chronic obstructive pulmonary disease. Am. J. Respir. Crit. Care Med..

[7] De Matteis, S. et al. The occupations at increased risk of COPD: analysis of lifetime job-histories in the population-based UK Biobank Cohort. *Eur. Respir. J*. **54**, 1900186 (2019). 10.1183/13993003.00186-2019 31248951

[8] National Institute for Health and Care Excellence (NICE). *Chronic Obstructive Pulmonary Disease In Over 16s: Diagnosis And Management* (2018). 31211541

[9] Haute Autorité de Santé (HAS). *Guide Du Parcours De Soins Bronchopneumopathie Chronique Obstructive* (2019).

[10] Quach A (2015). Prevalence and underdiagnosis of airway obstruction among middle-aged adults in northern France: The ELISABET study 2011-2013. Respir. Med..

[11] Bouet, P. *Approche Territoriale Des Spécialités Meédicales Et Chirurgicales (Situation au 1er Janvier 2021)* (Conseil National de l’Ordre des Medecins, 2021).

[12] Derom E (2008). Primary care spirometry. Eur. Respir. J..

[13] Moulis G (2015). French health insurance databases: what interest for medical research?. Rev. Med. Interne.

[14] Haroon SM (2015). Effectiveness of case finding strategies for COPD in primary care: a systematic review and meta-analysis. NPJ Prim. Care Respir. Med..

[15] SCORE Santé. Indicateurs scores. [cited on 28 June 2022]. Available at: https://www.scoresante.org/sindicateurs.html (2022).

[16] Conseil national de l’Ordre des médecins. *La Démographie Médicale* (2021).

[17] Tollanes MC (2020). Spirometry in chronic obstructive pulmonary disease in Norwegian general practice. BMC Fam. Pract..

[18] Soumagne T (2020). Quantitative and qualitative evaluation of spirometry for COPD screening in general practice. Respir. Med. Res..

[19] Giraud V (2016). Feasibility of spirometry in primary care to screen for COPD: a pilot study. Int. J. Chron. Obstruct. Pulmon. Dis..

[20] Bunge L (2018). [Study of the feasibility of spirometry in general practice]. Rev. Mal. Respir..

[21] Bremond M (2021). Micro-phenomenological approach to explore the patient experience during an initial spirometry examination to diagnose COPD in general practice in France. BMJ Open.

[22] Millien, C. C., Chaput, H. & Cavillon, M. La moitié des rendez-vous sont obtenus en 2 jours chez le généraliste, en 52 jours chez l’ophtalmologiste. *Direction de la Recherche, des Etudes, de l’Evaluation et des Statistiques (DREES)* (2018).

[23] Katsimigas A, Tupper OD, Ulrik CS (2019). Opportunistic screening for COPD in primary care: a pooled analysis of 6,710 symptomatic smokers and ex-smokers. Int. J. Chron. Obstruct. Pulmon. Dis..

[24] Chapron, A. et al. Early detection of Chronic Obstructive Pulmonary Disease in primary care: a randomised controlled trial. *Br. J. Gen. Pract*. 10.3399/BJGP.2022.0565 (2023). 10.3399/BJGP.2022.0565 PMC10633669 37903640

