# Supplementary material for: Spirometry practice by French general practitioners between 2010 and 2018 in adults aged 40 to 75 years
Source: NPJ Prim Care Respir Med. 2023 Sep 30;33:33. doi: 10.1038/s41533-023-00352-9 (PMC10542800; doi:10.1038/s41533-023-00352-9)
Supplement: Supplementary file 1 — suplementary informtions [file 41533_2023_352_MOESM1_ESM.pdf]

Supplementary informations

Supplementary Information 1 : The annual number of spirometries performed in France, by general practitioners (GPs), before excluding "expert GPs" performing more than 60 spirometries per year

Year	Number of spirometry	Number of GPs	Mean annual number of spirometries	Standard deviation	Min	Max	Median	P90
**2010**	29491	952	30.98	66.43	1	817	8	81
**2011**	31190	1147	27.19	63.64	1	818	7	72
**2012**	34489	1397	24.69	59.56	1	892	6	64
**2013**	32923	1363	24.15	58.16	1	869	6	60
**2014**	32676	1385	23.59	53.80	1	553	6	62
**2015**	33740	1554	21.71	48.87	1	541	6	55
**2016**	34575	1694	20.41	46.30	1	517	6	49
**2017**	36496	1880	19.41	45.43	1	475	5	47
**2018**	37094	1990	18.64	43.49	1	403	5	44
**Overall**	302674	13362	22.65	53.01	1	892	6	58

GPs: general practitioners, Min: minimum value, Max: maximum value, P90: the 90th percentile of the distribution of values.

Supplementary Information 2 : Evolution of the proportion of patients undergoing spirometry in France between 2010 and 2018

**Number of patients that had one or more spirometries each year**									
**Year**	**2010**	**2011**	**2012**	**2013**	**2014**	**2015**	**2016**	**2017**	**2018**
**Total**	**5145**	**5763**	**7745**	**7082**	**6754**	**8449**	**9288**	**10345**	**10976**
**Rate of patients that had a spirometry (for every 100,000 inhabitants)**	19.45	21.56	28.63	25.86	24.34	30.18	32.93	36.43	38.42
**French population aged between 40 and 75 years***	26,454,977	26,735,731	27,053,647	27,385,281	27,747,572	27,994,134	28,207,385	28,395,894	28,568,337
**Data from the Institut national de la statistique et des études économiques (Insee).[24]									
